# Telehealth Use Following COVID-19 Within Patient-Sharing Physician Networks at a Rural Comprehensive Cancer Center: Cross-sectional Analysis

**DOI:** 10.2196/42334

**Published:** 2023-01-17

**Authors:** Liyang Yu, You-Chi Liu, Sarah L Cornelius, Bruno T Scodari, Gabriel A Brooks, Alistair James O'Malley, Tracy Onega, Erika L Moen

**Affiliations:** 1 Department of Biomedical Data Science Geisel School of Medicine at Dartmouth College Lebanon, NH United States; 2 Quantitative Social Sciences program Dartmouth College Hanover, NH United States; 3 Department of Medicine Dartmouth-Hitchcock Medical Center Lebanon, NH United States; 4 The Dartmouth Institute for Health Policy and Clinical Practice Dartmouth College Hanover, NH United States; 5 Department of Population Sciences University of Utah Salt Lake City, NH United States

**Keywords:** telehealth, rural cancer care, patient-sharing networks, network analysis, COVID-19, cancer care, telemedicine, oncology, oncologist, electronic health record data, health system, patient network

## Abstract

**Background:**

In response to the COVID-19 pandemic, cancer centers rapidly adopted telehealth to deliver care remotely. Telehealth will likely remain a model of care for years to come and may not only affect the way oncologists deliver care to their own patients but also the physicians with whom they share patients.

**Objective:**

This study aimed to examine oncologist characteristics associated with telehealth use and compare patient-sharing networks before and after the COVID-19 pandemic in a rural catchment area with a particular focus on the ties between physicians at the comprehensive cancer center and regional facilities.

**Methods:**

In this retrospective observational study, we obtained deidentified electronic health record data for individuals diagnosed with breast, colorectal, or lung cancer at Dartmouth Health in New Hampshire from 2018-2020. Hierarchical logistic regression was used to identify physician factors associated with telehealth encounters post COVID-19. Patient-sharing networks for each cancer type before and post COVID-19 were characterized with global network measures. Exponential-family random graph models were performed to estimate homophily terms for the likelihood of ties existing between physicians colocated at the hub comprehensive cancer center.

**Results:**

Of the 12,559 encounters between patients and oncologists post COVID-19, 1228 (9.8%) were via telehealth. Patient encounters with breast oncologists who practiced at the hub hospital were over twice as likely to occur via telehealth compared to encounters with oncologists who practiced in regional facilities (odds ratio 2.2, 95% CI 1.17-4.15; *P*=.01). Patient encounters with oncologists who practiced in multiple locations were less likely to occur via telehealth, and this association was statistically significant for lung cancer care (odds ratio 0.26, 95% CI 0.09-0.76; *P*=.01). We observed an increase in ties between oncologists at the hub hospital and oncologists at regional facilities in the lung cancer network post COVID-19 compared to before COVID-19 (93/318, 29.3%, vs 79/370, 21.6%, respectively), which was also reflected in the lower homophily coefficients post COVID-19 compared to before COVID-19 for physicians being colocated at the hub hospital (*estimate:* 1.92, 95% CI 1.46-2.51, vs 2.45, 95% CI 1.98-3.02). There were no significant differences observed in breast cancer or colorectal cancer networks.

**Conclusions:**

Telehealth use and associated changes to patient-sharing patterns associated with telehealth varied by cancer type, suggesting disparate approaches for integrating telehealth across clinical groups within this health system. The limited changes to the patient-sharing patterns between oncologists at the hub hospital and regional facilities suggest that telehealth was less likely to create new referral patterns between these types of facilities and rather replace care that would otherwise have been delivered in person. However, this study was limited to the 2 years immediately following the initial outbreak of COVID-19, and longer-term follow-up may uncover delayed effects that were not observed in this study period.

## Introduction

The COVID-19 pandemic resulted in the rapid uptake of telehealth in cancer centers across the United States and around the world [1-3]. Since then, the advantages to sustained telehealth integration in cancer care have become more fully recognized, including increased access to specialists across greater distances, reduced travel burden for patients, and improved synchronous communication between regional specialists and community health care teams [4,5]. Telehealth in cancer care delivery, or tele-oncology, refers to the delivery of clinical oncology services through audio and video communication platforms to remote patients including chemotherapy monitoring, pain and symptom management, and palliative care [6]. In addition to the benefit of reducing infection risk during the COVID-19 pandemic, patients with cancer and treating physicians have reported general satisfaction with telehealth use in cancer care delivery [7-10]. However, challenges associated with telehealth remain, such as patient access to technology, quality of communication, establishing rapport between a physician and a new patient, and reimbursement policies [11-13]. Although survey results early in the pandemic found that the majority of oncologists were either highly likely or somewhat likely to continue using telehealth for established patients after the COVID-19 crisis [8], use-based data on telehealth visits in a post–COVID-19 era is needed to determine how and where telehealth may be having more persistent impacts on cancer care delivery.

The use of telehealth among oncologists may not only affect the way they deliver care to their own patients but also the physicians with whom they share patients. Patient-sharing networks built from administrative health care data, in which 2 physicians are connected if they have clinical encounters with common patients, provide a novel lens through which to study the impact of telehealth uptake. Patient-sharing relationships have been shown to capture self-reported professional relationships between physicians [14]. Patient-sharing networks have provided insight into informal integration between primary care and specialists, coordination among patient care teams, and locally unique linchpin physicians [15-17]. There is significant potential for patient-sharing networks to measure the impact of new policies and innovative care delivery models on the way in which physicians work together to deliver health care to their patients. For instance, the potential for telehealth to improve access to geographically distant providers may be reflected in changes to the patient-sharing patterns for cancer care within and between health systems. Within a health system spanning several hospitals, we hypothesized that telehealth might facilitate referrals between physicians at the hub hospital and physicians at regional facilities. This may in part occur due to an increase in web-based cancer multidisciplinary team meetings via web platforms, which allow specialized clinicians to join meetings they may not have had access to prior to COVID-19 [18]. However, if telehealth essentially replaces care that would otherwise have been delivered in person, we would expect to see minimal changes in the patient-sharing patterns with the uptake of telehealth.

The objective of this study was to examine telehealth encounters for patients diagnosed with breast, colorectal, and lung cancer within the Dartmouth Health system, home to a rural National Cancer Institute comprehensive cancer center. We first sought to identify characteristics of oncologists associated with telehealth encounters post COVID-19. Then, since we hypothesized that telehealth may lead to increased referrals between geographically distant providers, we examined whether there was an increase in the likelihood of patient-sharing ties between oncologists practicing at the Dartmouth Cancer Center in Lebanon, New Hampshire, and oncologists at regional hospitals. On March 15, 2020, Dartmouth Health implemented immediate social distancing policies due to the COVID-19 pandemic. Using this date to partition clinical encounters observed in the electronic health record data, we assembled pre- and post–COVID-19 patient-sharing networks for breast, colorectal, and lung cancer. We then assessed whether the structure of the patient-sharing networks changed between these time periods with the rapid uptake of telehealth.

## Methods

### Study Setting

Data were collected from electronic health records within the Dartmouth Health system in northern New England. The health system is comprised of a hub hospital in Lebanon, New Hampshire, where the Dartmouth Cancer Center resides, along with 5 sites and 15 regional affiliates across New Hampshire and Vermont.

### Ethics Approval

This study was approved by the Dartmouth Health institutional review board (study 02001168). All analyses were performed according to institutional review board and data use agreements with Dartmouth Health regarding the use of electronic health record data for research.

### Data Sources and Study Cohort

Retrospective data on adult patients diagnosed with incident breast, colorectal, and lung cancer between January 1, 2018, and December 31, 2020, were identified from the institutional tumor registry. Patients aged younger than 18 years or older than 99 years at the time of diagnosis were excluded. For those patients meeting our cohort criteria, we linked to the EPIC electronic health records at Dartmouth Health to identify their clinical encounters from 3 months prior to 12 months following their cancer diagnosis or through September 2021, whichever came first.

### Assembly of Patient-Sharing Physician Networks

To assemble pre–COVID-19 and post–COVID-19 patient-sharing networks for breast, colorectal and lung cancers, clinical encounters were stratified by pre- or post–COVID-19 time periods depending on whether the visit took place prior to March 15, 2020. Patient-sharing networks for each cancer type were assembled where 2 physicians were connected in the network if they had clinical encounters in the same time period with the same cancer patient.

### Study Variables

Physician characteristics of interest were specialty; patient volume; practicing in multiple locations; and practicing at the Dartmouth Health “hub” hospital in Lebanon, New Hampshire, home to the Dartmouth Cancer Center main campus. Physician specialty was obtained from electronic health record data. Cancer specialties included medical oncology, radiation oncology, general surgery, surgical oncology, plastic surgery for breast cancer, and thoracic surgery for lung cancer, where the latter 4 were collapsed into 1 category of surgery. Using encounters specific to either the pre- or post–COVID-19 time period, a physician was labeled as practicing at multiple sites if they had encounters in more than one ZIP code and as a “hub” hospital practitioner if they had clinical encounters with patients at the Dartmouth Health facility in Lebanon, New Hampshire. Patient characteristics included as covariates in the models included patient age in years at diagnosis and patient sex.

### Outcome Variable

The encounter-level outcome variable of interest was whether an encounter with an oncologist occurred via telehealth, which was inclusive of video and audio-only encounters.

### Statistical Analysis

Characteristics of patients and oncologists were summarized with descriptive statistics for each cancer type. Hierarchical logistic regression models were developed to study associations between the encounter-level variable of telehealth use and study variables. Random intercepts for patient and oncologist were specified to account for the nesting of encounters within patients and oncologists. To estimate the proportion of variance explained by patients and oncologists, intraclass correlation coefficients (ICC) for the patient and oncologist random effects were calculated. For example, the ICC for patient random effect is calculated by taking the ratio of between-patient variance and the total variance obtained from the mixed model. Hierarchical models were performed using the *lme4* package in R software (R Foundation for Statistical Computing) [19].

### Network Analysis

Networks were analyzed using the *visNetwork* and *igraph* packages in R and visualized with the Frutcherman-Reingold layout [20]. Global network statistics evaluated for the pre- and post–COVID-19 networks include density (the number of observed ties divided by the total number of possible ties), transitivity (the tendency of sets of 3 physicians to form a connected triangle), average distance (the average number of steps along the network it takes to connect each pair of physicians), and degree centralization (the variation in the degree centrality across physicians). These global network measures were chosen because they reflect distinct aspects of the structure of connections within a network. Prior work has indicated that patient-sharing networks with greater density have been associated with higher costs and use of services [21], and greater transitivity has been associated with patient-reported measures of care coordination [22]. Average distance was included to capture whether network paths between pairs of physicians became shorter or longer with the uptake of telehealth. Centralization was chosen because we hypothesized that if telehealth led to more care being coordinated between the hub hospital and regional facilities, it may lead to less care being concentrated among highly connected hub-hospital physicians, resulting in lower centralization. Edges between oncologists were labeled based on whether both, one, or none of the oncologists in the nonnull dyad practiced at the hub hospital. The proportions of each type of edge were calculated for the pre- and post–COVID-19 time periods.

Exponential-family random graph models (ERGMs) are probability models in which the network as a whole is the dependent variable that offer a flexible approach for handling the complex dependence structure of network graphs [23]. ERGMs are based on exponential-family theory for specifying the probability distribution for a set of random graphs or networks to describe the local selection forces that shape the global structure of the network [24]. Homophily describes the tendency of nodes in the network to form ties with similar others, and we were particularly interested in estimating homophily based on physician practice location. We estimated separate ERGMs for each time period (before and post COVID-19) to estimate the homophily coefficient for practicing at the hub hospital, which represents the change in the log-odds of the tie if the oncologists have the trait in common (either both practice at the hub hospital or both practice at regional facilities) compared to if they do not have the trait in common (a tie spanning an oncologist at the hub hospital and an oncologist at a regional facility), conditioned on the rest of the network. We present results for the exponentiated homophily term adjusted for the “edges” term (ie, density), so that the homophily coefficients represent the differences in the likelihood of edges existing between oncologists with the concordant level of location compared to oncologists in different locations using the *ergm* package in R [25].

## Results

Our study included patients with breast (n=1535), colorectal (n=601), and lung (n=1145) cancer (Table 1). The median age at diagnosis was 63, 66, and 68 years for patients with breast, colorectal, and lung cancer, respectively. Patients were 96.3% (3158/3281) White, which is reflective of the racial composition of northern New England. Of the total cohort of 3281 cancer patients, 951 (29%) patients had one or more telehealth encounters, and 939 of those patients were diagnosed post COVID-19.

The total number of oncologists across the 3 cancer types was relatively unchanged before and post COVID-19 (119 and 114, respectively), and 64.9% (74/114) of oncologists used telehealth post COVID-19 (Table S1 in Multimedia Appendix 1). Characteristics of oncologists by cancer type in the pre- and post–COVID-19 networks are summarized in Table 2. Telehealth use among oncologists before COVID-19 was rare (1%-3%), yet post–COVID-19 telehealth use was observed for 69% (53/77) of oncologists in the breast cancer network, 50% (33/66) of oncologists in the colorectal cancer network, and 61% (33/54) of oncologists in the lung cancer network. Of the 12,559 encounters between patient and oncologists post COVID-19, 1228 (9.8%) were via telehealth. The proportion of encounters with oncologists via telehealth and the number of oncologists using telehealth by month post COVID-19 peaks in April 2020 and then again around December 2020 (Figure S1 in Multimedia Appendix 1). Aside from telehealth use, none of the other characteristics of oncologists summarized in Table 2 were significantly different at *P*<.05 before and post COVID-19.

We found that the oncologist characteristics associated with telehealth encounters in the post–COVID-19 time period varied by cancer type (Table 3). Surgeons were less likely to have encounters via telehealth, and this association was statistically significant for breast cancer care (odds ratio [OR] 0.38, 95% CI 0.20-0.71; *P*=.003; reference=medical oncology). Additionally, for breast cancer care, patient encounters with oncologists with a medium patient volume were over 3 times as likely to occur via telehealth compared to patient encounters with low-volume oncologists (OR 3.84, 95% CI 1.09-13.62; *P*=.04), and patient encounters with hub hospital–based oncologists were over 2 times as likely to occur via telehealth compared with patient encounters with oncologists who practiced at regional facilities (OR 2.21, 95% CI 1.17-4.15; *P*=.01). For colorectal cancer care, we did not observe any significant associations between oncologist characteristics and telehealth encounters (*P*<.05); however, male patients with colorectal cancer were about half as likely to have telehealth encounters compared with female patients (OR 0.53, 95% CI 0.35-0.81; *P*=.003). For lung cancer care, patient encounters with radiation oncologists were more likely to occur via telehealth (OR 5.42, 95% CI 1.44-20.45; *P*=.01; reference=medical oncology), and patient encounters with physicians who practiced at more than 1 location were less likely to occur via telehealth (OR 0.26, 95% CI 0.09-0.76; *P*=.01). We assessed the variance in telehealth use explained by the random effects for patient and oncologist using the ICC (Table 3). The proportion of variance explained by the random effect for patient ranged from 0.14 for colorectal cancer to 0.17 for breast cancer. The proportion of variance explained by the random effect for oncologist ranged from 0.14 for colorectal cancer to 0.33 for lung cancer.

**Figure 1:**
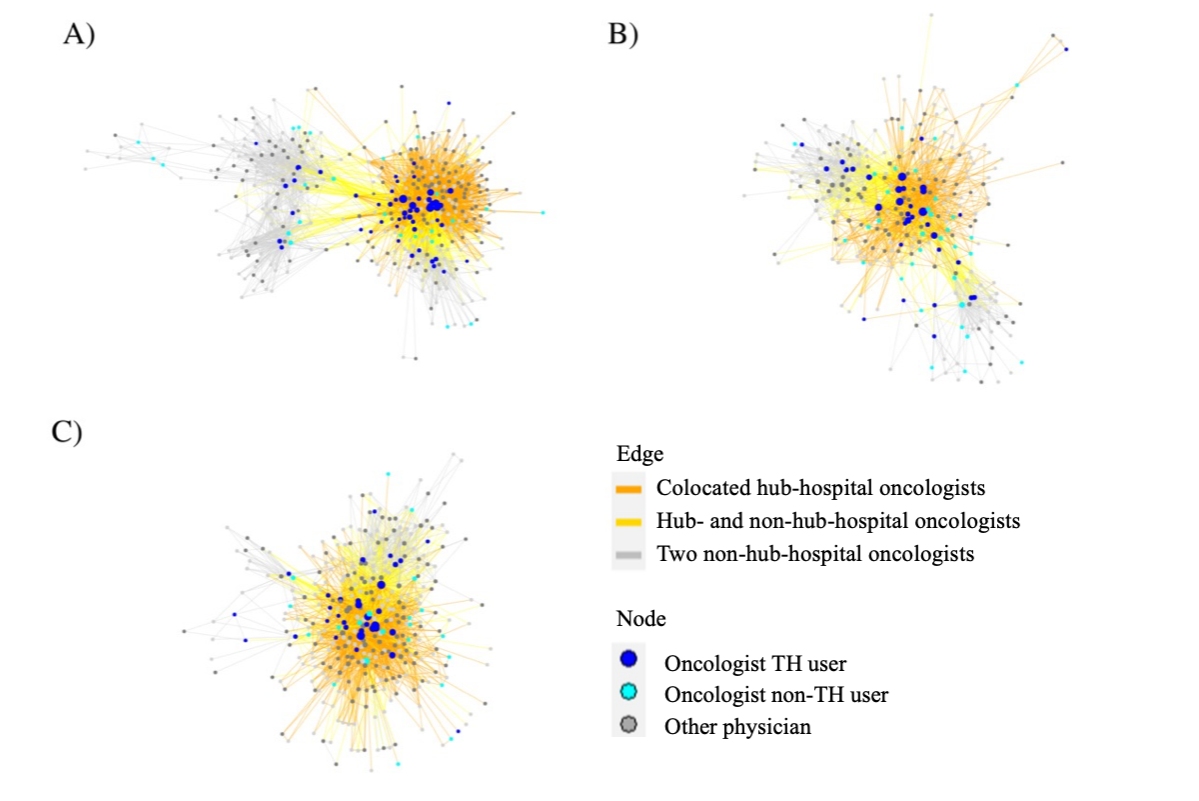
Post–COVID-19 patient sharing networks for (A) breast cancer, (B) colorectal cancer, and (C) lung cancer. TH: telehealth.

To gain insight into how telehealth use may have impacted relationships between oncologists, we assembled patient-sharing networks for cancer for the pre- and post–COVID-19 time periods. The post–COVID-19 patient-sharing networks for breast, colorectal, and lung cancer care are illustrated in Figure 1. Each node (circle) represents a physician, and a line connecting 2 nodes indicates that the 2 physicians shared patients with cancer. We assessed the overall structures of the pre–COVID-19 and post–COVID-19 networks based on 4 global network measures: density, the number of observed ties divided by the total number of possible ties; transitivity, the tendency of sets of 3 physicians to form a connected triangle; average distance, the average number of steps along the network it takes to connect each pair of physicians; and centralization, the variation in the degree centrality (number of ties) across physicians (Table 4). Network density, transitivity, and average distance were similar before and post COVID-19 for all 3 cancer networks. Centralization was lower in the post–COVID-19 network for all 3 cancer types, which may reflect less care being centralized among oncologists at the hub and more dispersed across providers in regional facilities.

We hypothesized that telehealth uptake in this health system may have led to more ties between pairs of oncologists that span the hub hospital and regional facilities in the post–COVID-19 network compared to the pre–COVID-19 network. Lung cancer care saw the most significant change in the distribution of edges across the 2 time periods, with 21.6% (79/370) of ties before COVID-19 and 29.3% (93/318) of ties post COVID-19 being between a hub hospital and non–hub hospital–based oncologist (*P*=.03; Table 4). For breast and colorectal cancer care, the distribution of ties was not statistically different between time periods. We next used *ergms* to examine the likelihood of ties forming between oncologists who are colocated at the hub hospital. In each cancer type, the homophily coefficient was positive in both time periods, reflecting the greater likelihood of a tie forming between pairs of physicians who both practice at the hub hospital (Table 4). If there is a greater likelihood of ties between pairs of oncologists that span the hub hospital and regional facilities post COVID-19 (conditional on the rest of the network), we would expect to see a lower homophily coefficient in the post–COVID-19 network compared with the pre–COVID-19 network. The most notable change before and post COVID-19 was observed in the lung cancer network. Ties between colocated oncologists were 2.45 (95% CI 1.98-3.03) times as likely to occur compared with those that were not colocated at the hub hospital in the pre–COVID-19 network, and the estimated likelihood in the post–COVID-19 network was reduced to 1.92 (95% CI 1.46-2.51).

**Table 1:** Characteristics of patients diagnosed with breast, colorectal, or lung cancer at Dartmouth Health.

Patient characteristic		Cancer type		
		Breast (n=1535)	Colorectal (n=601)	Lung (n=1145)
**Year of diagnosis, n (%)**				
	2018	514 (33.5)	208 (34.6)	396 (34.6)
	2019	531 (34.5)	199 (33.1)	411 (35.9)
	2020	490 (31.9)	194 (32.3)	338 (29.5)
Age at diagnosis (years), median (IQR)		63 (54-71)	66 (55-75)	68 (62-75)
Male, n (%)		<11^a^	300 (49.9)	556 (48.6)
**Race/ethnicity, n (%)**				
	Non-Hispanic White	1470 (95.8)	572 (95.2)	1116 (97.5)
	Non-Hispanic Black	17 (1.1)	<11	<11
	Hispanic/Latino	25 (1.6)	<11	<11
	Unknown	23 (1.5)	14 (2.3)	15 (1.3)
Ever used telehealth, n (%)		456 (29.7)	178 (29.6)	317 (27.7)

^a^Values with fewer than 11 patients were suppressed to protect patient confidentiality.

**Table 2:** Characteristics of oncologists in the pre- and post–COVID-19 patient-sharing networks.

Characteristic		Patient-sharing network type										
		Breast cancer				Colorectal cancer				Lung cancer		
		Before COVID-19 (n=78)	Post COVID-19 (n=77)	*P* value	Before COVID-19 (n=72)		Post COVID-19 (n=66)	*P* value	Before COVID-19 (n=77)		Post COVID-19 (n=54)	*P* value
**Specialty, n (%)**												
	Medical oncology	33 (42)	29 (38)	.83	35 (49)		33 (50)	.99	36 (47)		27 (50)	.50
	Radiation oncology	10 (13)	10 (13)		9 (12)		8 (12)		10 (13)		10 (19)	
	Surgery	35 (45)	38 (49)		28 (39)		25 (38)		31 (40)		17 (31)	
Patient volume, median (IQR)		16 (6-51)	10 (4-30)	.07	6 (3-14)		4 (2-11)	.10	6 (2-29)		9 (1-22)	.47
Multisite physician, n (%)		24 (31)	19 (25)	.40	17 (24)		16 (24)	.93	22 (29)		19 (35)	.42
Hub-hospital physician, n (%)		44 (56)	39 (51)	.47	45 (62)		41 (62)	.96	56 (73)		39 (72)	.95
Ever used telehealth, n (%)		2 (3)	53 (69)	<.001	1 (1)		33 (50)	<.001	2 (3)		33 (61)	<.001

**Table 3:** Multilevel models of the odds of an encounter being via telehealth by cancer type post COVID-19.

					Cancer type										
			Breast			*P* value		Colorectal		*P* value		Lung		*P* value	
**Patient characteristics,** OR^a^ (95% CI)															
	Age at diagnosis (years)			1.01 (0.99-1.02)			.24		1.00 (0.99-1.02)		.59		1.01 (0.99-1.03)		.25
	Male sex			N/A^b^					0.53 (0.35-0.81)^c^		.003		1.12 (0.80-1.57)		.51
**Oncologist characteristics**, OR (95% CI)															
	**Cancer specialty**														
		Medical oncology	Ref^d^					Ref				Ref			
		Radiation oncology	0.81 (0.30-2.21)			.69		0.73 (0.25-2.15)		.57		5.42 (1.44-20.45)^c^		.01	
		Surgery	0.38 (0.20-0.71)^c^			.003		0.49 (0.21-1.15)		.10		0.68 (0.18-2.58)		.57	
	**Patient volume**														
		Low	Ref					Ref				Ref			
		Medium	3.84 (1.09-13.62)^c^			.04		1.24 (0.38-4.07)		.74		1.98 (0.32-12.14)		.46	
		High	1.09 (0.40-2.96)			.87		0.92 (0.37-2.28)		.84		0.34 (0.09-1.28)		.11	
	Multisite physician			0.58 (0.28-1.22)			.15		0.70 (0.30-1.63)		.40		0.26 (0.09-0.76)^c^		.01
	Hub-hospital physician			2.21 (1.17-4.15)^c^			.01		1.10 (0.51-2.38)		.82		1.46 (0.45-4.72)		.53
**Intraclass correlation coefficient**															
	Oncologist			0.163			N/A		0.144		N/A		0.333		N/A
	Patient			0.171			N/A		0.135		N/A		0.142		N/A
	Overall			0.334			N/A		0.286		N/A		0.475		N/A

^a^OR: odds ratio.

^b^N/A: not applicable.

^c^Significant, *P*<.05.

^d^Ref: reference.

**Table 4:** Patient-sharing networks before and post COVID-19. Only nonnull dyads were considered when characterizing edges based on hub-hospital physicians.

				Before COVID-19		Post COVID-19		*P* value	
**Breast cancer**									
	**Global network statistics**								
		Density	0.057		0.044		N/A^a^		
		Transitivity	0.252		0.266		N/A		
		Average distance	2.229		2.478		N/A		
		Centralization	0.612		0.442		N/A		
	**Hub-hospital oncologists in dyad (before COVID-19: n=651; post COVID-19: n=635), n (%)**								
		2	369 (56.7)		353 (55.6)		.09		
		1	223 (34.3)		201 (31.7)				
		0	59 (9.1)		81 (12.8)				
	Homophily coefficient for practicing at the hub hospital (95% CI)		2.46 (2.08-2.9)^b^		2.74 (2.27-3.3)^b^		N/A		
**Colorectal cancer**									
	**Global network statistics**								
		Density	0.049		0.042		N/A		
		Transitivity	0.260		0.290		N/A		
		Average distance	2.330		2.602		N/A		
		Centralization	0.450		0.320		N/A		
	**Hub-hospital oncologists in dyad (before COVID-19: n=340; post COVID-19: n=339), n (%)**								
		2	215 (63.2)		190 (56.1)		.09		
		1	91 (26.8)		99 (29.2)				
		0	34 (10.0)		50 (14.8)				
	Homophily coefficient for practicing at the hub hospital (95% CI)		2.67 (2.14-3.33)^b^		2.55 (1.98-3.28)^b^		N/A		
**Lung cancer**									
	**Global network statistics**								
		Density	0.050		0.041		N/A		
		Transitivity	0.246		0.279		N/A		
		Average distance	2.198		2.424		N/A		
		Centralization	0.564		0.363		N/A		
	**Hub-hospital oncologists in dyad (before COVID-19: n=370; post COVID-19: n=318), n (%)**								
		2	277 (74.9)		208 (65.4)		.03		
		1	79 (21.6)		93 (29.3)				
		0	14 (3.8)		17 (5.4)				
	Homophily coefficient for practicing at the hub hospital (95% CI)		2.45 (1.98-3.03)^b^		1.92 (1.46-2.51)^b^		N/A		

^a^N/A: not applicable.

^b^Exponential-family random graph model.

## Discussion

This study assessed telehealth use within the Dartmouth Health system in rural northern New England. We found that physician specialty, patient volume, practicing at multiple locations, and practicing at the hub hospital were associated with telehealth use, but the strength of these associations differed across cancer types. Our study also corroborates recent work that found that variation across practices and clinicians explains a substantial amount of the variance in telehealth use [25,26]. Interestingly, we observed that patient encounters with oncologists who practice at more than 1 location were less likely to occur via telehealth. Considering telehealth and traveling oncologists are both potential levers that health systems can pull to increase access to care for otherwise underserved patient populations, examining how these resources complement each other and their impact on care quality and patient experience may inform strategies on equitable resource allocation to optimize access to care.

In addition to assessing characteristics of oncologists, our study examined how the uptake of telehealth post COVID-19 may have impacted the structure of relationships between physicians. Whether telehealth in oncology provides avenues for new referral paths and in what context will inform how this technology may be leveraged to address barriers in access to care in areas with limited oncologist supply. Patient-sharing networks showing significant changes, such as those we observed in the lung cancer network, may reflect new referral patterns between geographically distant providers that were established with the uptake of telehealth, whereas no changes may indicate that telehealth was primarily used in place of care that would have been delivered in person prior to the pandemic. This hypothesis could be explored in interviews with cancer providers and patients and tested further in larger claims-based data sets.

Our study has several limitations that may limit generalizability of our findings. First, all clinical encounters were limited to a single health care system. Dartmouth Cancer Center is the only National Cancer Institute–designated cancer center in northern New England, and its catchment area spans New Hampshire, Vermont, and some portions of Maine and northern New York. However, we are unable to observe clinical encounters that occurred outside of Dartmouth Health and its affiliated sites. Second, our study cohort only includes patients who were diagnosed through December 2020, and the data analyzed include their encounters 12 months following their diagnosis or through September 2021. The decline in telehealth use among oncologists observed for the last months of our study is likely exaggerated due to not including data from patients diagnosed with cancer in 2021. Efforts to track telehealth use among oncologists early versus late in the pandemic, and beyond 2021 as the data become available, may uncover associations and trends that were not evident in the time period analyzed in this study. Third, patient-level unmeasured confounders, such as insurance type and travel distance to physicians, may have contributed to telehealth use and the presence of ties in the patient-sharing network. Fourth, we are unable to control for other secular trends in the pre- and post–COVID-19 time periods that may have contributed to changes in the patient-sharing networks. Finally, this was a retrospective observational study so causality cannot be determined.

After the rapid uptake of telehealth in response to the COVID-19 pandemic, we are now starting to observe how and where telehealth may persist in the delivery of cancer care. Ongoing research on patient preferences and access to telehealth, in addition to physician and care team preferences, will be critical to mitigate disparate access to telehealth services [27-31]. Our study finds that the characteristics of oncologists who used telehealth differed across cancer types, indicating that efforts to standardize use across cancer care providers may be needed to reduce unwarranted variation in its implementation. Alternatively, our findings may indicate that the acceptability and appropriateness of telehealth varies across cancer types. The dissemination of guidelines for telehealth use during cancer care will support efforts aiming to reduce unwarranted variation in telehealth use among patients diagnosed with cancer and their care teams [32,33]. The extent to which telehealth changes access to cancer care and coordination of care among all providers and individuals in a patient’s care team is an area of active study [34]. Analyzing patient-sharing networks from administrative data as more current data sets become available can continue to shed light on whether telehealth is having an impact on cancer referral pathways and the organization of relationships between providers involved in cancer care.

## Appendix

Multimedia Appendix 1 Supplementary material.

## Abbreviations

ERGM: exponential-family random graph modeling
ICC: intraclass correlation coefficient
OR: odds ratio

## Data Availability

The data sets generated and analyzed for the current study are not publicly available to due data use agreements with Dartmouth Health because they contain protected health information. Researchers interested in obtaining these data would need to submit their own requests with Dartmouth Health Human Research Protection Program institutional review board.
