# Supplementary material for: Telehealth Use Following COVID-19 Within Patient-Sharing Physician Networks at a Rural Comprehensive Cancer Center: Cross-sectional Analysis
Source: JMIR Cancer. 2023 Jan 17;9:e42334. doi: 10.2196/42334 (PMC9848440; doi:10.2196/42334)

Telehealth use following COVID-19 within patient-sharing physician networks in a rural comprehensive cancer center: cross-sectional analysis

Appendix

Table S1. Characteristics of oncologists in the pre- and post-COVID patient sharing networks

Figure S1. Telehealth use post-COVID. (A) Proportion of encounters with oncologists via telehealth each month; (B) Number of oncologists using telehealth each month.

Table S1. Characteristics of oncologists in the pre- and post-COVID patient sharing networks

|  | **All** | |
| --- | --- | --- |
| Characteristic | Pre-COVID  N = 119 | Post-COVID  N = 114 |
| **Specialty**, n (%) |  |  |
| Medical oncology | 49 (41) | 45 (39) |
| Radiation oncology | 10 (8.4) | 10 (8.8) |
| Surgery | 60 (50) | 59 (52) |
| **Patient volume,** median (IQR) | 31 (4, 78) | 22 (4, 48) |
| **Multi-site physician**, n (%) | 31 (26) | 27 (24) |
| **Hub hospital physician**, n (%) | 87 (73) | 79 (69) |
| **Ever used telehealth**, n (%) | 4 (3.4) | 74 (65) |

IQR = interquartile range.

Figure S1. Telehealth use post-COVID. (A) Proportion of encounters with oncologists via telehealth each month; (B) Number of oncologists using telehealth each month.


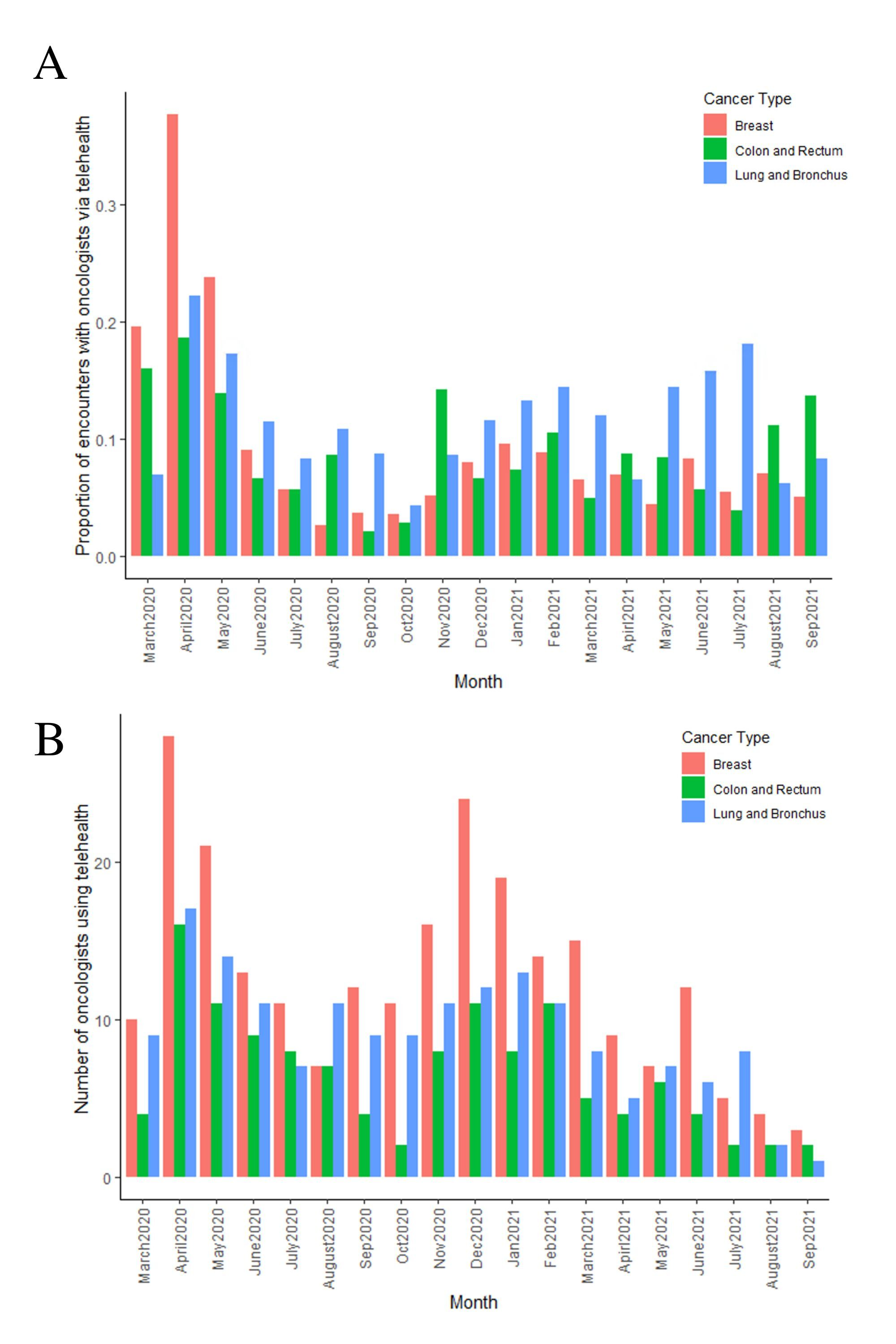

Supplement: Multimedia Appendix 1 [file cancer_v9i1e42334_app1.docx]
